# Micelle-Based Adjuvants for Subunit Vaccine Delivery

**DOI:** 10.3390/vaccines3040803

**Published:** 2015-09-25

**Authors:** Thomas Trimaille, Bernard Verrier

**Affiliations:** 1 Aix-Marseille Université, CNRS, ICR UMR 7273, 13397 Marseille, France; 2 Université Lyon 1, CNRS, LBTI UMR 5305, 69367 Lyon, France; E-Mail: bernard.verrier@ibcp.fr

**Keywords:** micelles, antigens, amphiphilic copolymers, peptide amphiphiles

## Abstract

In the development of subunit vaccines with purified or recombinant antigens for cancer and infectious diseases, the design of improved and safe adjuvants able to efficiently target the antigen presenting cells, such as dendritic cells, represents a crucial challenge. Nanoparticle-based antigen delivery systems have been identified as an innovative strategy to improve the efficacy of subunit vaccines. Among them, self-assembled micellar nanoparticles from amphiphilic (macro)molecules have recently emerged as promising candidates. In this short review, we report on the recent research findings highlighting the versatility and potential of such systems in vaccine delivery.

## 1. Introduction

Historically, vaccines have consisted of attenuated or killed microorganisms. However, due to their complex nature such vaccines can vary widely in quality from batch to batch and moreover can induce adverse effects. Recent advances in genomics and proteomics provided a wide variety of potential target antigens, including recombinant proteins, synthetic peptides or DNA [1]. While these clearly defined antigens offer important safety advantages, they often have a short halflife *in vivo* and are not always presented efficiently to the immune system, when administered alone without adjuvants. Until recently, hydroxide and phosphate salts of aluminum and calcium, and among them alum, were the only adjuvants licensed for human use [2]. But alum is a moderate adjuvant for antibody production, not suitable for all antigens, and is not efficient for promoting cell-mediated immunity. Consequently, considering the crucial issues in the field of cancer and infectious diseases research, significant research efforts have been devoted over the last decade to the development of new, improved vaccine adjuvants, such as emulsions [3], liposomes [4], or nanoparticles [5]. Among the latter, micelles are core-shell nanoparticles generated by spontaneous self-assembly in water of individual amphiphilic (hydrophobic/hydrophilic) molecules. Micellar systems are intrinsically interesting, in that thermodynamically driven self-assembly phenomena control the assembly shape and size as functions of the absolute and relative sizes of the hydrophobic and hydrophilic blocks. Historically used as drug delivery vehicles, through encapsulation/protection of hydrophobic drugs in the micelle core [6], micellar nanoparticles have been explored over the recent years as very valuable adjuvants for vaccine delivery. They indeed present interesting features over non-micellar adjuvant systems: (i) due to their small size (generally <100 nm) they particularly facilitate the antigen delivery to antigen presenting cells (APCs), such as dendritic cells (DCs) in the draining lymph nodes. These micelles indeed do not limit to association with DCs from the injection site, but are capable of targeting DCs by traveling through lymphatics directly to lymph nodes, where DCs are in greater concentration than in the periphery, promoting germinal center formation [7]; (ii) they can also easily display suitable surface properties (nature, surface charge) through the appropriate choice of biocompatible hydrophilic segments of the micelle corona. Surface properties of the carrier have been indeed identified as an important parameter regarding induction of immune responses [8]. For example, the “accelerated blood clearance” phenomenon, triggering the immune system, has been highlighted over the recent years [9,10,11], which may impact vaccine efficiency; (iii) moreover, through appropriate chemical design of the hydrophobic and hydrophilic blocks (presence of reactive groups, cationic moieties,…), a variety of additional immunostimulatory molecules (such as Toll Like Receptor (TLR) ligands, mannose receptor ligands,…) can be easily incorporated in these systems in a controlled fashion, to induce an enhanced activation of the DCs, which play a pivotal role in the immune responses.

**Figure 1:**
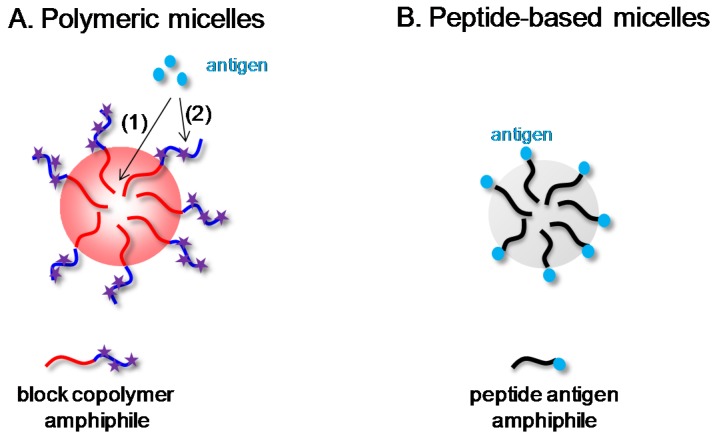
Schematical representation of the micellar nanoparticles developed as vaccine adjuvants; (**A**) polymeric micelles obtained by self-assembly of amphiphilic block copolymers in water, in/on which the antigenic peptide is encapsulated (1) or surface coupled (2) (reactive groups represented by star symbols); (**B**) micelles obtained from self-assembly of peptide antigen amphiphiles in water.

Basically two main types of micellar nanocarriers have been designed over the last years as adjuvant vaccines. In the first one, amphiphilic (*i.e.*, hydrophobic-hydrophilic) block copolymers are used for self-assembly into micelles while an antigenic peptide is associated to the latter through either encapsulation (1) or surface coupling (2) (Figure 1A). In the second one, also called “peptide amphiphile” self-adjuvant system, the antigenic peptide is used as the hydrophilic head-group and covalently bound to a hydrophobic moiety (typically a hydrophobic alkyl tail) for self-assembly into micelles (Figure 1B). In this short review, we describe the advances achieved with both adjuvant micellar systems.

## 2. Polymeric Micelles

### 2.1. Polylactide (PLA) Based Micelles

Polylactide (PLA) based nano- and micro-particles have been widely explored as safe adjuvants due to suitable PLA biodegradability and biocompatibility [12,13]. Based on this promising PLA polymer platform, Jimenez-Sanchez *et al.* have recently designed micelles from a PLA-b-P(*N*-acryloxysuccinimide-co-*N*-vinylpyrrolidone) (PLA-b-P(NAS-co-NVP)) block copolymer [14]. While an antigenic protein (HIV-1 Gag p24) could be surface coupled to the micelle corona through reaction of its lysine and N-terminal amines with the N-succinimidyl pendant groups of the NAS units, a hydrophobic TLR ligand (imiquimod) was encapsulated in the PLA core of the micelles. An enhanced *in vitro* stimulation/maturation of the DC was observed with the encapsulated imiquimod as compared to free analog, and these immunostimulatory properties of the loaded imiquimod were shown to be conserved when the p24 antigen was coupled at the micelle surface. These data regarding improved immunostimulatory efficiency suggested the strong potential of this micelle-based nano-system for vaccine delivery [14]. 

In other works, Jain *et al.* compared the immunogenicity of hepatitis B surface antigen (HBsAg) formulated by PLA polymer or poly(ethylene glycol)-PLA-poly(ethylene glycol) (PEG-PLA-PEG) block copolymer [15]. The results showed that PEG-PLA-PEG micelles were much more potent than PLA nanoparticles to enhance and prolong HBsAg-induced mucosal antibody responses through both intranasal and oral immunization [15,16].

### 2.2. Polypeptide Based Micelles

Interesting studies were devoted to micelles based on polypeptides, using both hydrophilic and hydrophobic poly(amino-acid) sequences.

The group of Ma has developed micelles based on poly(ethylene glycol)-b-poly(l-lysine)-b-poly (l-leucine) (PEG-PLL-PLLeu), creating an hydrophobic core of PLLeu, an intermediate layer of PLL and outer shell of PEG [17]. The polypeptide micelles could simultaneously encapsulate ovalbumin (OVA) and polyriboinosinic: polyribocytidylic acid (PIC), a TLR3 agonist, through electrostatic interactions between the cationic PLL and negatively charged OVA and PIC, to synergistically augment tumor specific cytotoxic-T-lymphocyte (CTL) response. In a cancer vaccine context, to overcome the tumor associated DC (TADC) dysfunction (*i.e.*, poor responsiveness upon TLR stimulation), related to the hyperactivity of the STAT3 (signal transducer and activator of transcription 3), this team has encapsulated in the micelles a STAT3 siRNA [18] together with PIC and OVA. These micelles not only successfully attenuated STAT3 signaling in TADCs but also effectively induced TADC maturation both *in vivo* and *in vitro.*

Polymer micelles based on hydrophilic poly(γ-glutamic acid) (γ-PGA) bound to hydrophobic cholesterol moieties were also developed and found to provide efficient mucus delivery and induce antigen-specific high mucosal immunity, in a perspective of needle-free vaccine [19]. The carboxy groups of the γ-PGA can act as a mucoadhesive in the presence of mucus layer glycoproteins whereas partial polymer modification with amine moieties allows interaction with the anionic epithelial cell layer. The authors tested the adjuvant function of the γ-PGA micelles in presence of the influenza A viral antigen (PR8). They found that intranasal immunization with PR8 in the presence of the micelles induced high levels of PR8-specific IgG titers in the sera of mice as well as mucosal IgA antibody titers in their nasal washes, as compared to immunization with PR8 alone. They also found that PR8 plus γ-PGA micelles were able to elicit high levels of IFN-γ producing cells, showing the micelle system could function as an effective adjuvant for inducing both humoral and cellular responses. Furthermore, the mice immunized with PR8 plus γ-PGA micelles exhibited 100% protective immunity to the lethal PR8 virus challenge, as compared to 50% for the mice immunized with only PR8.

With a similar micellar system, based on self-assembly of γ-PGA polymer modified with hydrophobic l-phenylalanine ethylester, DC activation/maturation was shown to be size dependent, the effect of particles size on DC maturation being stronger for the smaller particles [20]. In further studies, intranasal immunization with these poly(γ-PGA) nanoparticles entrapping antigenic proteins was shown to induce potent tumor immunity [21]. It was finally shown that these nanoparticles increased vaccine efficiency in a single dose when co-administered with virus-like particles [22].

Hao *et al.* have reported an innovative micellar construction based on PEG-b-PLL block copolymer [23]. The ε-amine of PLL was used for coupling of thiopyridyl disulfide groups able to react with an antigen bearing four cysteines. As a result, the micelles were composed of a PLL/peptide based crosslinking core and a PEG outer shell. Immunostimulatory DNA (ISS-DNA) was also complexed in the PLL cationic core through electrostatic interactions. Interestingly, after phagocytosis, the antigen and ISS-DNA can be released through reduction of the disulfide crosslinks in the presence of high intracellular gluthathione concentration. The antigen can be then processed in the APCs and presented to T cells, while the released ISS-DNA can induce the APCs to secrete the cytokines needed for effective T cell activation and proliferation. Cell culture studies demonstrated that these peptide crosslinked micelles greatly enhance the uptake of peptide antigens into human DCs.

### 2.3. pH Responsive Micelles

pH-responsive micelles, based on a block copolymer poly(*N*-(2-hydroxypropyl) methacrylamide-co-pyridyl disulfide methacrylamide)-b-poly(acrylic acid-co-dimethylaminoethyl methacrylate-co-butyl methacrylate) (P(HPMA-co-PDSMA)-b-P(AA-co-DMAEMA-co-BMA)), were also developed [24]. The presence of carboxylic groups from the acrylic acid units in the polymeric core can indeed impart an endosomolytic activity. The presence of the pyridyl disulfide groups on the micelle corona allowed the reversible conjugation of cysteine containing ovalbumin (OVA) antigen. Interestingly, mechanistic studies in a murine dendritic cell line (DC 2.4) demonstrated micelle-mediated enhancements in intracellular antigen retention and cytosolic antigen accumulation. Conjugation of OVA to the micelles significantly enhanced antigen cross-presentation *in vitro* relative to free OVA, an unconjugated physical mixture of OVA and polymer, and a non-pH responsive micelle-OVA control. Also, the pH-responsive carrier facilitated antigen delivery to APCs in the draining lymph nodes. Finally, Subcutaneous immunization of mice with OVA-polymer conjugates significantly enhanced antigen-specific CD8+ T cell responses (0.4% IFN-γ+ of CD8+) compared to immunization with soluble protein, OVA and polymer mixture, and the control micelle without endosome-releasing activity. The authors have also further shown the beneficial impact of introducing a CpG oligodeoxynucleotide (CpG ODN, binding TLR9) onto the micelles (through electrostatic interactions with cationic moieties on the micelle corona) on the immune response [25]. 

Boudier *et al.* have also reported pH-sensitive micelles based on polymethacrylic acid-b-polyethylene glycol/poly-L-lysine (PLL) for antigen peptide delivery [26]. The *in vitro* study showed that these polyion complex micelles not only effectively loaded antigen peptides, but also facilitated their uptake and release in DCs. Moreover, these polyion complex micelles significantly promoted DC maturation, indicating their immunostimulatory effect [26,27].

### 2.4. Other Polymeric Micelles

The group of Hubbell developed a PEG-b-poly(propylene sulfide) block copolymer, able to self-assemble in 25–35 nm micelles, and whose PEG extremity bears a pyridyl disulfide moiety to enable coupling of cysteine containing OVA antigen [28]. Biodistribution studies showed that these micelles were able to travel to draining lymph nodes, where they preferentially interacted with APCs. When mice were immunized in conjunction with free CpG as an adjuvant, OVA-conjugated micelles generated more (2.4-fold) OVA-specific CD8+ T cells in the blood and higher (1.7-fold) interferon gamma levels from splenocytes upon restimulation than in mice immunized with free OVA and CpG.

Other studies were performed on cationic polyethyleneimine (PEI) based micelles. The hydrophobic stearic acid was covalently bound to PEI to generate micelles consisting of a PEI corona and stearic acid based core able to encapsulate an hydrophobic melanoma peptide antigen Trp2 (SVYDFFVWL sequence) [29]. After subcutaneous injection into mice, Trp2-loaded micelles accumulated preferentially in the medulla and paracortex of the draining lymph nodes and were present at negligible levels in the systemic circulation. Mice immunized with Trp2-loaded micelles showed significantly higher Trp2-specific cytotoxic T lymphocyte activity than mice immunized with free Trp2 or a mixture of Trp2 and empty micelles. In a B16-F10 murine melanoma model, Trp2-loaded micelles inhibited tumor growth significantly more than did free Trp2.

### 2.5. DNA Vaccination with Polymeric Micelles 

The DNA vaccination approach using micellar adjuvants has been also considered. Very recently, Layek *et al.* focused on DNA vaccination against Hepatitis B virus (HBV) using micelles based on chitosan modified with hydrophobic phenyl alanine [30]. Mannose moieties were also introduced on the polymer to trigger mannose-receptor mediated endocytosis in APCs. The “polyplexes” obtained through electrostatic interactions between anionic DNA (GFP expressing plasmid) and cationic moieties (protonated amines) of the polymer micelles showed enhanced cell uptake (RAW 264.7 and DC 2.4 cells) and high *in vitro* transfection efficiency. Moreover, intradermal immunization of BALB/c mice indicated that polyplexes with the plasmid DNA encoding hepatitis B surface antigen (pHBsAg) induced significantly higher serum antibody titer in comparison to naked pHBsAg, and non-mannosylated polyplexes. Most importantly, they triggered potent cellular immune response, which is crucial for the eradication of HBV infected cells. In order to induce both antibody production and T-cell activity, Yang *et al.* have designed a DNA fragment containing multiple-epitope antigen gene (MA) of HCV with five conserved epitopes (three T-cell epitopes and two B-cell epitopes), which was delivered in PLA-PEG-PLA block copolymer micelles [31]. Satisfying immune responses were achieved after single immunization. Successful DNA vaccination experiments were also reported with a block copolymer composed of poly{*N*′-[*N*-(2-aminoethyl)-2-aminoethyl]aspartamide} (P(Asp)DET) and PEG, in which the p(Asp)DEP polycationic part is able to complex plasmid DNA to form polyplex micelles with a PEG corona, protecting the DNA in the inner core [32,33]. The polyplex micelles exhibited high transfection efficiency and enhancement of the endosome escape [34]. When administered intraperitoneally, the polyplexes with encapsulated tumor-associated antigen SART3, CD40L and GM-CSF plasmid genes significantly prolonged the survival of mice harboring peritoneal dissemination of CT26 colorectal cancer cells. 

In recent years, messenger RNA (mRNA)-based vaccines have emerged to be an interesting alternative to DNA-based vaccines due to the safety of not inserting into host genome. Using PEI-stearic acid based micelles mentioned above [29], the team of Sum has protected anionic HIV-1 gag RNA by complexation with the cationic PEI corona of the micelles [35]. The micelle/mRNA complexes could significantly enhance anti-HIV-1 gag immune responses as compared to mRNA alone and PEI-mRNA complex, resulting in high antigen-specific antibody secretion and pro-inflammatory cytokines production, which make this strategy a possible anti-HIV-1 vaccine candidate.

## 3. Peptide Amphiphiles

Another interesting micellar construct consists of peptide amphiphiles (PAs). PAs consist of a hydrophobic, lipid-like tail linked to a hydrophilic, biofunctional antigenic peptide headgroup. Under aqueous conditions, the PAs self-assemble into micelles in which the tails are buried in the core away from water, and the antigenic peptides are displayed on the outside (Figure 1B).

The group of Tirrell has designed a molecule consisting of a dialkyl tail with two palmitic (C16) chains, conjugated to a peptide containing the known cytotoxic T-cell epitope from the model tumor antigen OVA [36]. The Tc-cell epitope sequence, residues 257–264 (SIINFEKL), was elongated to include residues 253–266 in order to increase the hydrophilic character of the peptide for self-assembly purposes and to increase the length of the peptide to aid in processing by DCs, for suitable presentation of peptide antigens to T-cells. After subcutaneous immunization on mice that were inoculated with cancer cells genetically engineered to express OVA, the tumors grew significantly slower in mice that received the diC16-OVA micelles compared to either the control PBS group or the peptide in Incomplete Freund adjuvant (IFA) group. Mice that were immunized with diC16-OVA survived longer than those immunized with PBS or the OVA peptide in IFA.

In a similar approach [37], the same team has developed peptide amphiphiles comprising of a group A streptococcus B cell antigen (J8) and the dialkyl hydrophobic moiety, organized into self-assembled micelles. When injected into mice, the micelles induced a strong IgG1 antibody response that was comparable to soluble J8 peptide supplemented with classical adjuvants such as IFA [37]. 

In other studies, Accardo *et al.* have also designed micelles based on peptide amphiphiles as vaccines to treat Herpes simplex virus (HSV) [38]. The amphiphiles consisted of peptide epitopes selected from the HSV envelope glycoproteins B and D (gB and gD), that had their N-terminus modified with hydrophobic moieties containing a double C18 alkyl chain. The cytokine production triggered by the gB and gD micelles in mouse (RAW 264.7) and human (U937) macrophages increased compared to the production triggered by the pure gB and gD peptides. 

The group of Irvine [39] has also developed such type of amphiphilic peptides (amph-peptide), as well as amphiphilic CpG (amph-CpG) containing a hydrophobic albumin binding lipid tail, that promote efficient accumulation in the lymph nodes. Using a model HIV peptide antigen (AL11 epitope from SIV Gag), the tumor-associated self-antigen Trp2 from melanoma, and a peptide derived from the human papillomavirus (HPV)-derived cervical cancer antigen E7, they showed that mice immunized with amph-peptides and amph-CpG showed markedly increased expansion of antigen-specific, cytokine-producing CD8+ T-cells and enhanced cytolytic activity relative to unmodified peptide/CpG immunizations. In addition, in animals bearing established TC-1 tumors (expressing the E7 oncoprotein from HPV) or B16F10 melanomas, vaccination with the amph-peptides/amph-CpG triggered sustained regression of large TC-1 tumors that were only modestly affected by soluble vaccines and slowed the growth of melanoma tumors, in which a traditional soluble vaccine had no effect.

Other studies focused on peptide amphiphiles with a dendritic structure consisting of a hydrophobic polyacrylate core and a peripheral generation of a peptide antigen (J14, B cell epitope on the streptococcal M protein) [40]. The dendrimer structure resulted in a self-assembled micellar nanoparticle of 20 nm diameter in water. Following subcutaneous immunization in mice, the nanoparticles produced high levels of systemic J14-specific IgG antibody similar to that observed with the injection of J14 epitope with complete Freund’s adjuvant as a positive control. Significantly higher antibody titers were observed for the nanoparticles conjugated with J14 epitopes in comparison with a physical mixture of J14 with pure polyacrylate dendrimer core, thus suggesting that chemical conjugation of epitopes with a polymer core was essential to elicit an immune response. 

Zope *et al.* have recently described peptide amphiphiles composed of an hydrophobic poly(γ-benzyl-l-glutamate) covalently linked to a cell penetrating peptide, namely TAT peptide [41]. Its arginin-rich amino acid sequence led to micelles with a positive surface charge, which was used to adsorb negatively charged influenza hemaglutinin antigen (HA) as well as negatively charged CpG oligonucleotide as an immunostimulatory molecule. The co-delivery of CpG and HA with this vehicle resulted in an enhanced immune response in mice against the HA antigen, towards a Th1 response. This formulation also demonstrated a higher immunogenicity when compared with the commonly used adjuvant Al(OH)_3_.

## 4. Conclusions and Outlooks

Over the recent years, micelles structures have been found to represent a highly suitable and tunable platform for vaccine delivery as illustrated through numerous proofs of concepts. Indeed, through an appropriate and flexible chemical design (tunable size, appropriate surface charge and/or reactive groups for antigenic peptide/immune-stimulatory molecule immobilization, pH-sensitivity, *etc.*), these systems are able to display highly attractive and controllable properties having a beneficial impact on the efficiency of the immune response and its orientation. Indeed, they could deliver with high efficiency either nucleic acids (DNA, RNA) or proteins moieties, and a wide range of immune molecules, through their highly versatile backbone design. Even if there still exists room for improvement for transforming micelles as vaccine vehicles ready for human use, they definitively appear to be challenging candidates to emulsion or to particulate vaccine delivery platforms.
